# No evidence that lionfish *Pterois miles* coordinate and reciprocate during hunts

**DOI:** 10.1098/rsos.220834

**Published:** 2023-02-15

**Authors:** Hanaa Sarhan, Redouan Bshary

**Affiliations:** Institution of Biology, University of Neuchatel, Emile-Argand 11, 2000 Neuchatel, Switzerland

**Keywords:** coordinated hunting, reciprocate hunts, alternation, lionfish *Pterois miles*

## Abstract

Decision rules underlying cooperative hunting can range from very simple to very complex. As lionfishes are naturally solitary hunters, an experimental study documenting active recruitment, coordination and alternating (potentially reciprocal) striking in dwarf lionfish *Dendrochirus zebra* received major attention. A hypothesis was that sophisticated coordinated hunting may also contribute to the successful invasion of another lionfish species, *Pterois miles*, in the Caribbean. However, we did not find recruitment signalling in *P. miles* in parts of its native range, the Red Sea. Here, we expand on these results, testing for coordinated movements and for alternation in strikes**.** We exposed subject pairs to inaccessible prey in three transparent housings. The two lionfish did not aggregate at the same prey housing or even share larger space units in the presence of prey. In a second experiment, we found that some alternation can be induced if prey items become alternately accessible at two corners, with each lionfish tending to monopolize one corner each. When the movement of prey is slow or even absent, we observed less alternation than expected by chance. In conclusion, *P. miles* in the Red Sea does not use any coordination to hunt prey.

## 1. Introduction

Cooperative hunting occurs when two or more individuals interact in a hunt that produces higher payoffs per individual than solitary hunting [1]. Coordinated cooperative hunting, in which individual predators respond in time and space to each other's actions, is considered to be a complex form of cooperative hunting [2,3]. It has been observed and described in primates [4,5], carnivores [3,6,7], marine mammals [2,3,8], birds [9] and fishes [10]. In its most complex form, a collaborative hunting, individuals adopt specific hunting roles to herd and catch their prey [2,3,6]. Complexity can be further increased if hunting partners actively communicate with each other, for example to initiate a hunting event [4,10].

Given that complex forms of cooperative hunting are rare and proposed to be linked to specific ecological challenges, one would not expect evidence for complex cooperative hunting in predators like lionfish, which typically hunt alone [11–13], though they have been rarely observed to hunt in the presence of conspecific lionfishes [14] or moray eels [15]. However, the hunting behaviour of lionfish *Dendrochirus zebra* (Scorpaenidae: Pteroinae) described in laboratory experiments [16] was very sophisticated. More precisely, *D. zebra* subjects swam away from an inaccessible shoal of prey fish (behind a glass barrier) to join a partner initially out of sight. There, subjects repeatedly showed a stereotyped fin flaring pattern that was interpreted as a recruitment signal to elicit joint hunting with both conspecifics and another lionfish species *Pterois antennata*. The signal recipient would join the signaller and return to the prey that was made accessible by the experimenter. Both lionfish would then simultaneously herd prey by spreading their own pectoral fins and alternate feeding strikes at the shoal [16]. The alternation of strikes was interpreted as reciprocal acts to ensure sharing of prey in a cooperative task. While *D. zebra* only occurs in the Indo-Pacific, *P. antennata* is one of the two *Pterois* species invasive to the Caribbean [17,18]. It was therefore hypothesized that cooperative hunting may be an important part of lionfish invasion success [16].

While concerns were raised about the credibility of the published data, as sample sizes were lower than declared [19] and attempts were made to hide this fact with a collage of pictures [20], knowing the hunting strategies of *Pterois* species may potentially contribute to understanding their invasion success in the Caribbean, and more recently the Mediterranean [21–23]. In a first study on *Pterois miles* in the Red Sea, no evidence was found that individuals seek each other's presence in the field [13]. Furthermore, the stereotyped fin flaring movement pattern was also observed in *P. miles*, but it was not associated with signalling situations. The authors concluded that fin flaring is linked to manoeuvring in tight places [13], which would also have applied to the area in front of the partner in the study by Lönnstedt *et al*. [16].

Here, we expand on [13] and investigate two main questions using laboratory experiments. First, we asked to what degree two *P. miles* individuals coordinate in space and time when exposed to three alternative hunting locations, i.e. glass aquaria of identical sizes spread out inside the larger lionfish holding tank, each containing four prey fish. If *P. miles* individuals seek cooperative hunting, we predicted that pairs would aggregate at the same prey aquarium more than expected by chance. Second, we investigated to what extent and under which conditions two *P. miles* individuals alternate striking at prey. Lönnstedt *et al.* [16] had interpreted the alternation as reciprocity, i.e. individuals deciding to alternate as a cooperative solution to the competition over resources. We tested this hypothesis of reciprocity against an alternative hypothesis that alternations might be linked to the laboratory setting imposing constraints on monopolization. More specifically, in a rectangular tank as used in [16], it is conceivable that prey might aggregate in one corner when facing two lionfish, where the nearest lionfish conducts the first strike. The strike might make the surviving prey flee into the adjacent corner, where now the other individual is nearest and hence will strike, which will make surviving prey return to the first corner, and the circle restarts. Thus, alternation could be due to prey behaviour rather than based on reciprocity between lionfish. In order to test our hypothesis versus the reciprocity interpretation indicated by the alternation of feeding strikes and equality of food acquisition outcome, we created a ‘feeding tree’, a hand-held stick with several branches and a piece of food item (fresh tuna or prawn) on each end. The experimenter moved the stick through the aquarium, making single food items at a time accessible at specific locations in various predefined patterns that either hindered or facilitated monopolization by one fish. According to the reciprocity interpretation introduced in [16], our manipulations should not affect the alternation pattern of strikes (e.g. the equality and turn taking between individuals to take food items). Alternatively, some movement patterns should yield asymmetries in foraging success while others promote alternating strikes due to imposed constraints on individual monopolization.

## 2. Material and methods

### 2.1. Spatial coordination

#### 2.1.1. Fish handling and experiment set-up

**Figure 1.**
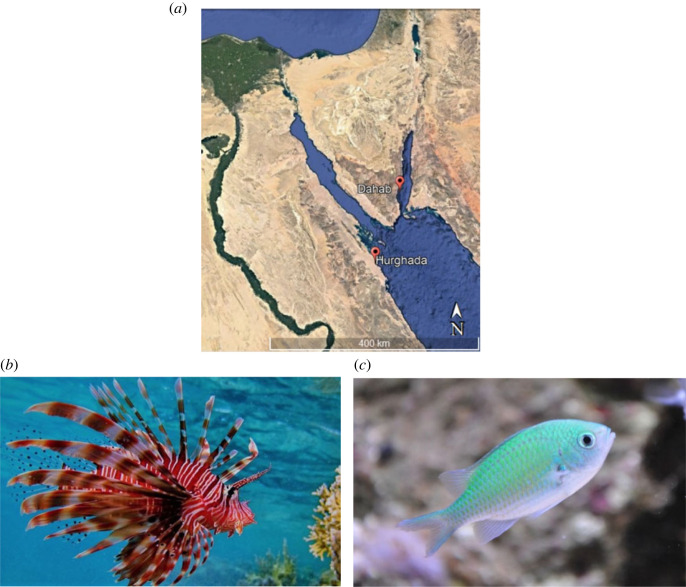
(*a*) Location of the conducted study in the northern Red Sea region. Image modified from Google Earth v. 6.2 (2021). (*b*) The study species *P. miles*. (Photo credit: Hanaa Sarhan). (*c*) The prey species *Chromis viridis* provided as bait during experiment trails. (Photo credit: Lubomír Klátil).

The coordination experiments were conducted in the Open Ocean Research Center (OOSC) located in Dahab, South Sinai, Red Sea, Egypt (28.5091° N, 34.5136° E), from May to June 2021 (figure 1*a*). Fish capturing, handling and acclimatizing process was performed carefully to minimize stress responses and to provide a neutral environment for the captured individuals. For lionfish capture, we built a plastic net fixed to a hand net frame (40 cm diameter) and gloves and captured 46 individuals varying in size from 20 to 29 cm total lengths via scuba diving with a slow approach from depths of 0 to 10 m. Individuals were brought slowly to the surface to avoid air bladder inflation. We assigned individuals directly into single pairs in each round tank and left them to acclimatize for 10 days. The round experiment tank was 153 cm diameter × 60 cm high with a water level of 40 cm. Starting from the third night, individuals were fed twice during the crepuscular time two pieces of dead shrimps per individual, while during experimental days, individuals were fed only after trials to ensure that they were motivated to hunt during trials. Blue-green chromis (*Chromis viridis*, figure 1*c*) were captured in parallel to present them as potential prey to lionfish. For blue-green chromis, we caught 25 individuals with average size of 4 cm using a barrier net of 1.5 × 1.5 m and 0.5 × 0.5 cm mesh size. Chromis were kept in a single aquarium (70 × 40 × 40 cm) and left to acclimatize for 7–10 days. An artificial coral block and small PVC tubes were added to serve as shelters, and blue background was placed all around the aquarium. Fish were fed twice per day with commercial flakes; food residuals were removed daily. We kept each species in a separate seawater system where aquaria were filled 2 days before fish accommodation, provided with seawater open system flow (2 l min^−1^), an air pump to provide appropriate aeration and water submersible pumps to provide current. All aquaria were cleaned once a day to prevent biotic accumulation. All fish were released at sites of capture after completing the experiment.

#### 2.1.2. Experimental protocol

**Figure 2.**
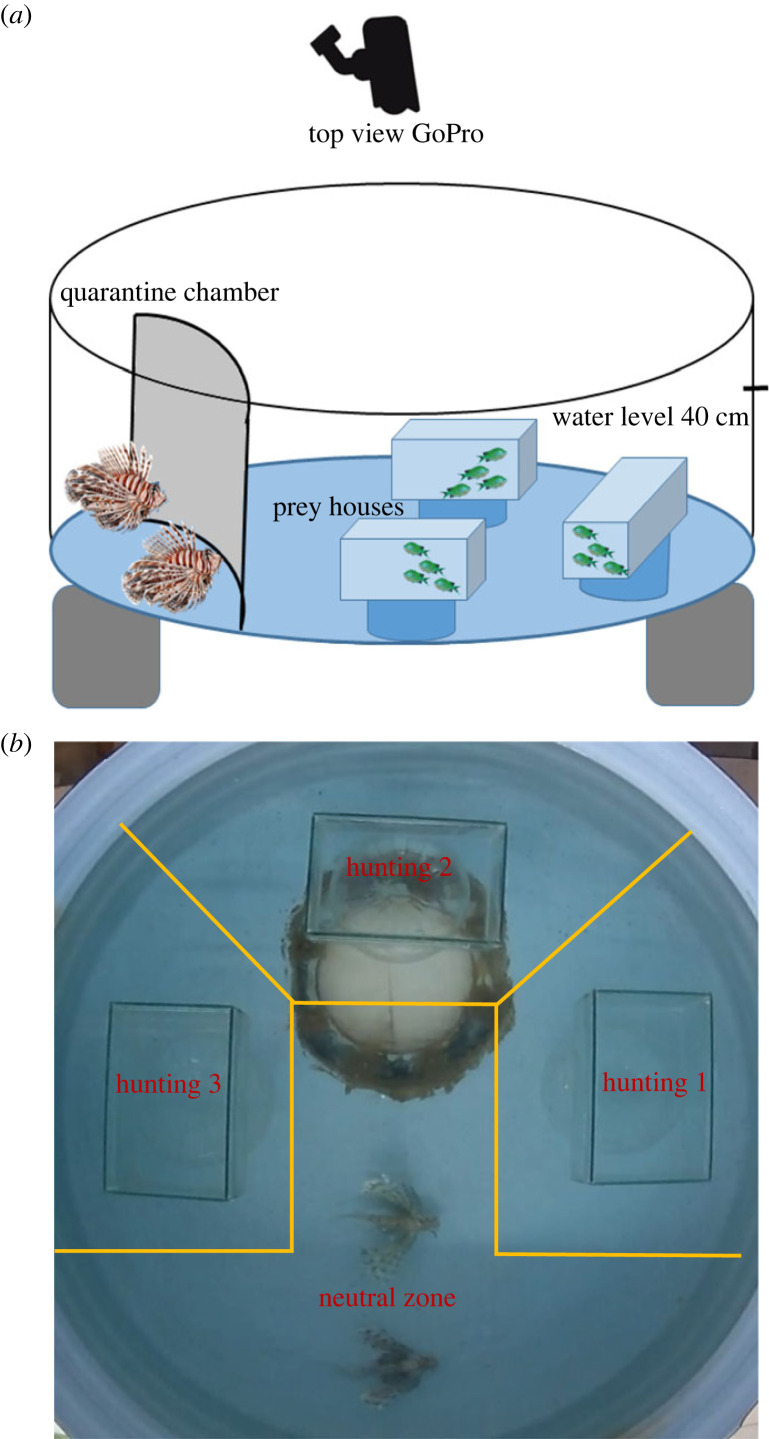
Tank set-up to test the coordination of lionfish partners. (*a*) Illustrating the tank structure and locations of instalments before starting the trial. (*b*) Live photo of experiment zones in control condition after partners was released from the quarantine.

The lionfish partners were first pushed slowly into an opaque compartment and isolated there for 15 min, while setting up the experiment. Three prey housing glass aquaria (30 × 15 × 25 cm) were placed in the tank with 45 cm space in between to allow subjects to move freely around them (figure 2*a,b*). After the prey housings were installed and prey individuals were assigned randomly to the prey housings (figure 2*a*), we let the fish settle for 10 min. Then, the partners were released simultaneously by lifting the opaque compartment and filmed for 10 min from above. Each lionfish pair was tested three times in each of the two conditions: (i) general prey absence and (ii) four prey individuals per housing aquarium. For data collection and analysis purposes, we divided the tank into four *zones:* three separate hunting zones around each of the three prey housing aquaria and a fourth ‘neutral zone’ away from prey (figure 2*b*).

### 2.2. Alternated strikes

#### 2.2.1. Fish handling and experiment set-up

**Figure 3.**
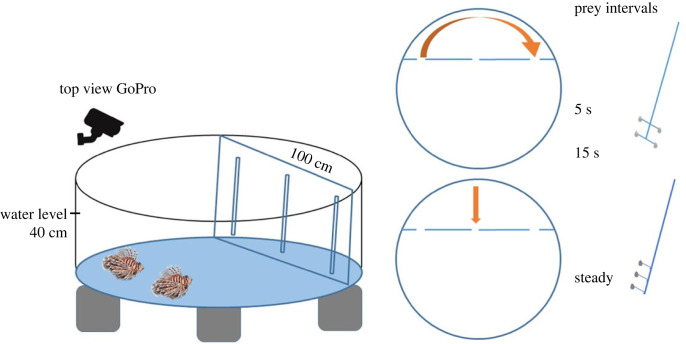
Tank set-up and types of bait used to investigate the alternation of lionfish partners. The bait and the three-movement pattern assigned for each bait.

The alternation experiments were conducted in Hurghada grand aquarium, Hurghada (27.1337° N, 33.8217° E) in March 2022. Lionfish sample individuals were collected in 2020–2022 and kept in the exhibition and in the hold tanks. Fishermen used cages to capture lionfish individuals to avoid harmful contact and transferred to the aquarium hospital tanks to be treated for potential injuries or infections, then let to acclimatize. After recovery, individuals were fed pieces of fresh tuna (1 × 1 cm) twice a day by throwing food items randomly in the tank. Twenty-five Lionfish individuals assigned to be tested were transferred in pairs to the round experimental tanks (153 cm diameter × 60 cm high, with a water level of 40 cm) one week prior to the experimental trials, matching individuals in size as much as possible. Training consisted of moving our ‘feeding tree’, a hand-held main stick with three or four branches to attach food items (figure 3), randomly in the tank, so that the two lionfish could simultaneously access it and hence compete over food. Within 3–4 days, all individuals followed the stick and ate food items off it. In this way, by moving the food tree selectively, individuals were fed twice during the crepuscular time periods two pieces of fresh tuna per individual. On the fifth day, experimental trials started.

#### 2.2.2. Experimental protocol

Like in the previous experiment, lionfish pairs were first confined to one side of the tank, behind an opaque barrier. We then placed a transparent barrier (100 × 60 cm) on the opposite side, creating an inaccessible compartment. That barrier had three vertical cuts 20 cm long and 3 cm wide, 30 cm apart from each other (figure 3). The experimenter could slide side arms of the feeding tree holding uni-sized pieces of tuna at predetermined schedules (time and location) through these cuts such that they became accessible to lionfish during trials. We conducted a single final training round in which three items were offered successively through each of the three slits, with the order counterbalanced across pairs. We did not score how many items each individual ate during that training. Then, the actual experiments began, consisting of three different treatments. As we were interested in the subjects' spontaneous behaviour, each pair was tested only once in each treatment. The order of treatments was counterbalanced across pairs to avoid that potential sequence effects could influence overall results. During the first two treatments, four items became successively accessible through the two side cuts. Crucially, accessibility alternated between the two cuts: as soon as one item was eaten on one side, the feeding tree was retracted and moved to the other cut, and moved back once a second item had been eaten, and finally again moved for the fourth food item to become accessible. The difference between the two treatments was the time the experimenter took to move the tree from one cut to the other: 5 s versus 15 s. The third treatment involved a feeding tree with only three branches and hence three food items. The branches were invariably introduced to the lionfish side through the middle cut, with all three branches being simultaneously accessible. In all three treatments, a trial started by having the feeding tree in its first accessible position and then lifting the opaque partition to release the partners simultaneously. A trial ended in principle when all pieces had been eaten. Only once, individuals had not eaten all items after 180 s exposure, and the feeding tree was removed. This data point was removed from the analysis. We recorded the order of attacks by individuals with a GoPro Hero + 3 camera attached to the tank side.

### 2.3. Statistical analysis

All statistical analyses were conducted with RStudio Inc., version 1.2.5033, and videos were analysed using BORIS software v. 7.9.7-2021. All the data are available as electronic supplementary material on Dryad: https://doi.org/10.5061/dryad.g79cnp5s8.

The aim of the first experiment was to investigate the level of coordination of lionfish in space and time to hunt their prey. For the analyses on spatial coordination, we extracted the following information separately for trials in which prey was present and trials in which prey was absent: (i) time in seconds from release until the two fish were in the same hunting zone for the first time, (ii) the time in seconds they would spent together in that zone before separating, and (iii) the overall time in seconds spent together in the same hunting zone, and overall time spent together in the neutral zone. The first two datasets were directly compared between trials in which prey was present and trials in which prey was absent. For the third dataset, we first quantified the time each individual spent in each of the four zones. The data allowed us to calculate an expected value for the two fish being simultaneously in each of the four zones based on the null hypothesis that the two fish move independently of each other (equation (2.1)). We then added up the three observed values for simultaneous presence in each of the three hunting zones, and we added up the three expected values, for a comparison. We also compared observed and expected values of co-occurrence in the neutral zone. Due to the repeated measures, pair IDs were added as a random variable. In general, we applied a linear mixed-effect model to investigate how treatment affected our variables of interest (nlme: ‘lme’).

2.1 $$\begin{array}{rl} & \mathrm{Expected}\;\mathrm{duration}\;\mathrm{to}\;\mathrm{overlap}\;\mathrm{in}\;\mathrm{zone}\;\mathrm{in}\;\mathrm{seconds}\\ & \;=\left(\frac{\mathrm{observed}\;\;\mathrm{in}\;\mathrm{zone}\;.\;\mathrm{ind}\;1\;\mathrm{in}\;s\;}{\mathrm{trial}\;\mathrm{duration}\;\mathrm{in}\;s}\right)\times \left(\frac{\mathrm{observed}\;\mathrm{in}\;\mathrm{zone}\;.\;\mathrm{ind}\;2\;\mathrm{in}\;s}{\mathrm{trial}\;\mathrm{duration}\;\mathrm{in}\;s}\right)\times \;\mathrm{trial}\;\mathrm{duration}\;\mathrm{in}\;s \end{array}$$

To test whether lionfish alternate strikes in the second experiment, we scored the number of switches in each trial (0–3 possible during the first two treatments, 0–2 possible during the third treatment). We then calculated the difference between the observed alternation count and expected alternation value based on the null hypothesis that individuals feed at random. To calculate the expected value of the null hypothesis that individual success is random for each item presentation, we divided the maximal possible number of switches for each trial by 2. As the first two treatments involved four food items, there were three occasions on which the successful individual could change or remain the same, each event with 50% probability. Hence, for the first two treatments (5 s and 15 s intervals), the expected value of the null hypothesis was 1.5 alternations, for the third treatment (steady) it was 1 alternation. As we faced many ties between observed values and the null hypothesis, we used a simple sign-test. We calculated the positive and negative ranks, which were used to extract the test statistics and *p*-value.

## 3. Results

### 3.1. Spatial coordination experiment

**Figure 4.**
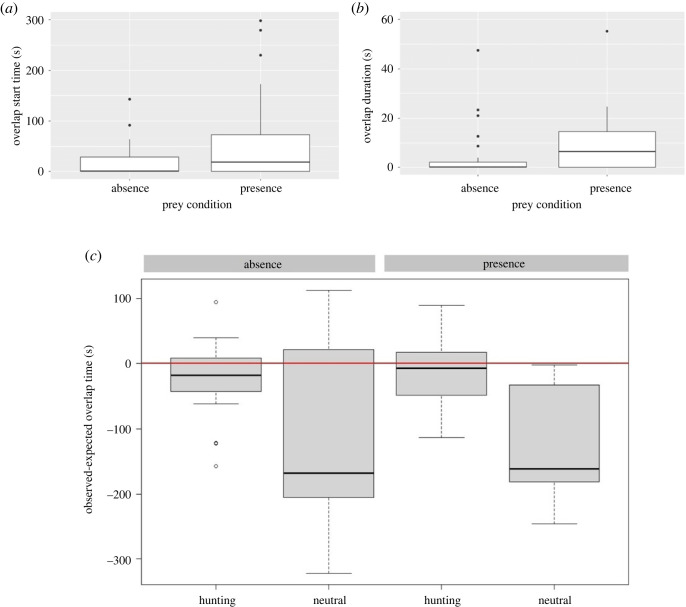
A comparison of time budget spent by *P. miles* in absence and absence of prey conditions. (*a*) The time spent by individuals till the overlap or associate for the first time at any of prey housings. (*b*) The duration of the first overlap by the paired individuals. (*c*) The expected time to be spent in association in each zone, compared with the actual observed time.

We had 230 min video material for 23 pairs. In the presence of prey, it took individuals longer to join each other for the first time in one prey zone (i.e. focusing on the same prey holding aquarium) compared with the treatment in which prey was absent (presence: mean = 55.68 ± 10.76 s, and absence: mean = 17.57 ± 5.42 s, *χ*^2^ = 4.354, d.f. = 1, *p* = 0.037, figure 4*a*). Once together, pairs tended to stay for longer in the same hunting zone when prey was present (presence: mean = 14.33 ± 6.42, and absence: mean = 4.50 ± 2.33, *χ*^2^ = 3.63, d.f. = 1, *p* = 0.056, figure 4*b*).

Overall, however, lionfish either ignored or tended to avoid each other during the trials. On a purely descriptive level, expected mean values of being together were higher than observed mean values in all four conditions (hunting zones/neutral zone × prey present/prey absent). The full model revealed a significant effect of location (*χ*^2^ = 24.6, d.f. = 1, *p* < 0.0001) but not of prey presence/absence (*χ*^2^ = 0.01, d.f. = 1, *p* = 0.91), and no interaction between the two factors (*χ*^2^ = 0.942, d.f. = 1, *p* = 0.331, figure 4*c*). *Post hoc* tests revealed the lionfish pair generally spent significantly less time together than expected in the neutral zone (prey present: *t* ratio = −5.27, *p* < 0.0001; prey absent: *t* ratio = −4.11, *p* = 0.0001). In the presence of prey, expected and observed values did not differ significantly (prey present: *t* ratio = −1.22, *p* = 023; prey absent: *t* ratio = −1.97, *p* = 0.054).

### 3.2. Alternated strikes experiment

Due to the deliberately short training period, we did not observe individuals focusing on any food insertion location. We did record that lionfish would distribute or separate themselves such that each individual is waiting at each slit in any of the 12 pairs. Between the insertion's duration, both partners followed the movement of the feeding tree on the other side of the transparent barrier. As a result, both individuals where typically near the feeding tree when a food item was made accessible through a slit. Keeping up with the food tree movement was simply more challenging when the interval was 5 s rather than 15 s.

**Figure 5.**
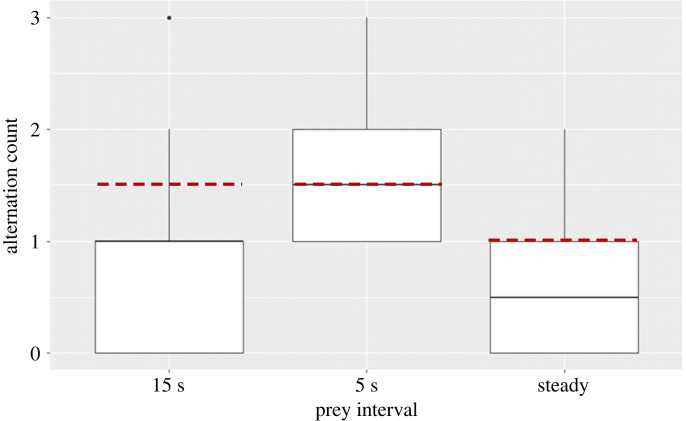
Boxplots of the number of observed alternations of strikes within trials versus the null hypothesis that alternations occur at random (red dashed lines). In the first two treatments, a total of four prey items was offered, alternating between two different locations. In the first treatment, time interval between prey item presentations was 15 s, in the second treatment it was 5 s. In the third treatment, three items were made accessible simultaneously at one location.

Using 12 pairs, we generally found that individuals did not alternate their strikes above chance levels. The amount of alternations apparently depended on the time interval between successive presentations during the first two treatments. The 5 s interval treatment yielded a random pattern (sign-test, *N* = 12, *X* = 6, *p* = 1). By contrast, the 15 s treatment yielded significantly less alternations than expected by chance (sign-test, *N* = 12, *X* = 2, *p* = 0.038, figure 5). Finally, in the steady treatment, observed alternations occurred less than expected by chance but not significantly so (sign-test, *N* = 12, 4 ties, remaining *N* = 8, *X* = 2, *p* = 0.29, figure 5).

## 4. Discussion

We had aimed to test whether lionfish *P. miles* inhabiting the Red Sea expressed any evidence of hunting in a coordinated way as a first step towards assessing the hypothesis that such coordination may contribute to their successful expansion in their invasive range in the Caribbean Sea. We did not find any evidence for coordination in time and space, nor for alternation in hunting strikes. Thus, we failed to replicate results reported on another lionfish species *D. zebra* in Australia. As a consequence, the literature on cooperative hunting and potentially underlying cognitive mechanisms, including signalling, coordination or reciprocity, does not apply to *P. miles* in the Red Sea.

Regarding coordination in space and time (first experiment), the presence of prey delayed lionfish pairing up in the same prey zone. While they then stayed together for longer than in the control, this result was apparently driven by both being independently attracted to the prey. If lionfish coordinated in space and time, we would have expected that they spent overall more time together in the same zones than expected by chance. If anything, the data suggest that lionfish tend to avoid each other and hence prefer to hunt alone. This result aligns with pre-existing field observation reporting that lionfish *P. miles* in nature are mostly solitary hunters [12,17,24]. It contrasts with the described coordination of movements in *D. zebra* [16]. Note, however, that the data are not directly comparable as only we offered alternative prey locations and quantified each individual's location over the entire trial.

Given that our lionfish did not coordinate in time and space, it may not be surprising that they did not alternate their strikes at prey as reported for *D. zebra* [16]. Importantly, one experimental design caused more alternating strikes, i.e. when fast movement of the feeding tree (a.k.a. prey shoal) from one end of the separation to the other allowed each individual to focus on a different gap. Thus, prey movement patterns could in principle cause alternating strikes in confined aquarium space, especially as prey would move faster between locations than our feeding tree. Unfortunately, the experimental videos of Lönnstedt *et al*. [16] are not available. Otherwise, re-analysing the videos would reveal whether prey indeed moved back and forth between corners, whether they thereby alternated their proximity to the two lionfish, and hence whether prey movement caused the reported alternations in strikes.

In summary, our previous [13] and the current study on *P. miles* fail to reproduce results on *D. zebra* [16], finding no evidence for active recruitment, coordination in time and space, or reciprocity-like alternation in strikes. Thus, lionfish are not in general sophisticated cooperative hunters. We therefore consider it parsimonious to assume that the success of the genus *Pterois* in the Caribbean [16,17] is linked to other features already documented.

## Data Availability

All data are available on Dryad: https://doi.org/10.5061/dryad.g79cnp5s8 [25]. The data are provided in the electronic supplementary material [26].
